# Supplementary material for: Bowhead whale faeces link increasing algal toxins in the Arctic to ocean warming
Source: Nature. 2025 Jul 9;644(8077):693–8. doi: 10.1038/s41586-025-09230-5 (PMC12367538; doi:10.1038/s41586-025-09230-5)
Supplement: Supplementary file 1 — Supplementary Tables 1–9. [file 41586_2025_9230_MOESM1_ESM.pdf]

nature portfolio

https://doi.org/10.1038/s41586-025-09230-5

Supplementary information

Bowhead whale faeces link increasing algal toxins in the Arctic to ocean warming

In the format provided by the authors and unedited

Supplementary Information

Table S1. Collection dates and algal toxin (domoic acid [DA] and saxitoxin [STX]) concentrations (ng/g) in bowhead whale fecal samples (n = 205). All whales with algal toxin concentrations below detectable limits of algal toxin assays were assigned "0 ng/g".

ID	Collection Date (m/d/y)	DA (ng/g)	STX (ng/g)
04-1	9/22/04	4	109
04-2	9/22/04	0	24
04-3	9/26/04	0	73
04-4	9/26/04	0	16
04-5	9/26/04	0	6
04-6	9/18/04	13	95
05-1	10/1/05	842	17
05-2	10/2/05	668	6
05-3	10/2/05	13	0
05-4	10/3/05	184	17
05-5	10/3/05	75	13
05-6	10/4/05	47	19
05-7	10/5/05	39	11
05-8	10/5/05	7719	19
06-1	9/29/06	6	0
06-2	9/29/06	6	21
06-3	9/29/06	32	39
06-4	9/30/06	28	69
06-5	9/30/06	1843	8
06-6	9/30/06	7	79
06-7	10/1/06	13	36
06-8	10/1/06	4	129
06-9	10/2/06	22	0
06-10	10/2/06	0	0
06-11	9/25/06	6	33
06-12	9/25/06	69	26
06-13	9/25/06	709	15
06-14	9/26/06	625	15
06-15	9/28/06	7	0
07-1	10/7/07	7	9
07-2	10/7/07	0	39
07-3	10/8/07	0	7
07-4	10/9/07	0	22
07-5	9/3/07	429	0
07-6	9/11/07	183	0
08-1	10/5/08	50	63
08-2	10/6/08	7	31
08-3	10/6/08	16	28
08-4	10/9/08	9	22
08-5	10/9/08	20	19
08-6	10/14/08	57	7
08-7	10/14/08	142	21
08-8	10/14/08	19	33
08-9	10/17/08	7	5
08-10	10/22/08	1073	40
08-11	10/23/08	414	41

09-1	9/27/09	8	25
09-2	9/29/09	7	22
09-3	9/29/09	0	10
09-4	10/6/09	0	162
09-5	10/10/09	0	39
09-6	10/10/09	0	102
09-7	9/26/09	0	207
09-8	9/26/09	0	276
09-9	9/27/09	0	10
09-10	9/14/09	126	12
10-1	10/7/10	52	6
10-2	10/7/10	103	184
10-3	10/8/10	798	22
10-4	10/9/10	12	15
10-5	10/9/10	137	298
10-6	10/11/10	18	42
11-1	10/24/11	0	4
11-2	10/24/11	0	6
11-3	10/27/11	108	10
11-4	10/27/11	114	0
11-5	10/29/11	1655	0
11-6	10/29/11	273	0
11-7	10/29/11	24	0
11-8	10/30/11	638	0
11-9	10/8/11	40	104
12-1	10/1/12	0	40
12-2	10/1/12	0	60
12-3	10/1/12	0	313
12-4	10/3/12	0	143
12-5	10/3/12	0	133
12-6	10/5/12	5	245
12-7	10/5/12	0	332
12-8	10/19/12	0	89
13-1	9/22/13	17	40
13-2	9/23/13	206	45
13-3	9/25/13	15	29
13-4	9/25/13	0	0
13-5	9/25/13	20	79
13-6	9/26/13	32	74
13-7	9/26/13	20	34
13-8	9/26/13	35	59
13-9	9/28/13	44	90
13-10	9/28/13	27	37
13-11	9/28/13	86	28
13-12	10/2/13	27	52
13-13	9/15/13	0	3
13-14	9/16/13	20	21
13-15	9/16/13	6	33
13-16	9/22/13	0	0
13-17	9/22/13	4	59
13-18	8/30/13	8	11
13-19	9/12/13	16	9
14-1	10/7/14	8	185
14-2	10/8/14	4	34
14-3	8/30/14	8599	13
14-4	9/1/14	387	12
15-1	9/25/15	0	36
15-2	9/25/15	0	18
15-3	9/25/15	0	31

15-4	9/27/15	0	5
15-5	9/27/15	8	29
15-6	9/27/15	0	41
15-7	9/28/15	0	34
15-8	10/2/15	0	100
15-9	10/2/15	0	50
15-10	10/2/15	0	81
15-11	10/3/15	0	70
15-12	10/3/15	0	41
15-13	10/4/15	9	91
15-14	10/4/15	51	54
16-1	9/20/16	65	76
16-2	9/22/16	689	20
16-3	9/24/16	1187	13
16-4	9/26/16	234	25
16-5	9/27/16	260	30
16-6	9/27/16	147	15
16-7	10/4/16	17	24
16-8	10/4/16	40	53
16-9	10/6/16	100	185
16-10	10/6/16	32	23
16-11	9/6/16	9	0
17-1	10/5/17	211	58
17-2	10/5/17	7531	52
17-3	10/5/17	13	147
17-4	10/8/17	4512	135
17-5	10/8/17	8	13
17-6	10/8/17	11	150
17-7	10/9/17	927	149
17-8	10/9/17	49	132
17-9	10/9/17	134	149
17-10	10/11/17	2119	44
17-11	10/11/17	15	75
17-12	10/11/17	6259	82
17-13	10/1/17	37	58
18-1	10/7/18	4	23
18-2	10/7/18	0	8
18-3	10/9/18	0	17
18-4	10/9/18	13	53
18-5	10/19/18	0	84
18-6	10/19/18	0	76
18-7	10/19/18	0	31
18-8	10/19/18	0	65
18-9	10/20/18	0	51
18-10	10/20/18	0	0
18-11	10/20/18	0	8
18-12	10/21/18	0	23
18-13	10/22/18	0	51
18-14	10/22/18	12	69
18-15	10/23/18	0	21
18-16	10/23/18	0	40
18-17	10/7/18	5	33
19-1	8/30/19	8606	3
19-2	9/4/19	1778	0
19-3	8/30/19	1872	3
20-1	8/25/20	0	16
20-2	8/28/20	0	27
20-3	8/29/20	0	23
20-4	8/30/20	0	22

20-5	9/4/20	0	24
20-6	9/4/20	0	20
20-7	9/4/20	0	8
20-8	9/5/20	0	8
20-9	9/8/20	0	17
20-10	9/8/20	0	12
20-11	9/17/20	0	17
20-12	9/17/20	0	25
20-13	10/11/20	0	13
20-14	10/12/20	0	16
21-1	8/25/21	40	0
21-2	9/28/21	131	81
21-3	9/28/21	185	59
21-4	10/3/21	0	46
21-5	10/4/21	82	65
21-6	10/4/21	3	76
21-7	10/6/21	0	60
21-8	10/7/21	0	0
21-9	10/7/21	0	67
21-10	10/7/21	0	45
21-11	10/9/21	0	47
21-12	10/9/21	0	42
21-13	10/9/21	0	48
21-14	9/9/21	13	15
21-15	9/14/21	0	0
21-16	9/16/21	0	0
22-1	10/1/22	0	5
22-2	10/1/22	36	18
22-3	10/1/22	57	48
22-4	10/2/22	9	55
22-5	10/2/22	0	17
22-6	10/2/22	0	30
22-7	10/2/22	47	41
22-8	10/3/22	21	17
22-9	10/4/22	0	18
22-10	10/4/22	0	10
22-11	10/4/22	0	11
22-12	10/4/22	0	12
22-13	10/4/22	0	15
22-14	10/5/22	0	18
22-15	10/5/22	12	24

Table S2. *Alexandrium catenella* cell densities through the water column along the Barrow Canyon East (BCE) transect (Fig. 1e) on August 20, 2019. (Note: The surface *A. catenella* cell density data depicted as part of Figure 1e were previously published in Anderson et al. 2021.

Station	Cast	Latitude	Longitude	Depth (m)	*A. catenella* (cells L^{-1})
BCE-1	107	71.5357	-154.0313	3.6	730
BCE-1	107	71.5357	-154.0313	9.9	917
BCE-1	107	71.5357	-154.0313	20.1	1750
BCE-1	107	71.5357	-154.0313	45	4
BCE-2	108	71.5957	-153.9502	3.2	1154
BCE-2	108	71.5957	-153.9502	9.9	1273
BCE-2	108	71.5957	-153.9502	19.9	375
BCE-2	108	71.5957	-153.9502	46.1	17
BCE-3	109	71.6498	-153.8398	3.3	990
BCE-3	109	71.6498	-153.8398	10.3	1173
BCE-3	109	71.6498	-153.8398	20.1	1358
BCE-3	109	71.6498	-153.8398	46.2	15
BCE-4	110	71.7147	-153.7393	3.6	259
BCE-4	110	71.7147	-153.7393	9.8	294
BCE-4	110	71.7147	-153.7393	19.9	513
BCE-4	110	71.7147	-153.7393	52.4	2
BCE-5	111	71.7815	-153.63	3	796
BCE-5	111	71.7815	-153.63	10.1	878
BCE-5	111	71.7815	-153.63	19.9	898
BCE-5	111	71.7815	-153.63	40	0
BCE-6	112	71.8387	-153.5562	2.9	177
BCE-6	112	71.8387	-153.5562	10.3	209
BCE-6	112	71.8387	-153.5562	20.2	17
BCE-6	112	71.8387	-153.5562	40.3	4
BCE-7	113	71.8928	-153.459	3.2	28
BCE-7	113	71.8928	-153.459	10.4	18
BCE-7	113	71.8928	-153.459	20.3	8
BCE-7	113	71.8928	-153.459	40.4	3
BCE-8	114	71.9517	-153.3652	3.2	7
BCE-8	114	71.9517	-153.3652	10	58
BCE-8	114	71.9517	-153.3652	20.2	12
BCE-9	115	72.0202	-153.2562	4	21
BCE-9	115	72.0202	-153.2562	10.2	7
BCE-9	115	72.0202	-153.2562	19.5	10

Table S3: Statistical results (unpaired two-sided t-tests with no *P*-value adjustments) of the monthly summer (June – September) estimated marginal mean (EMM) Beaufort Sea open water (OW) area anomaly comparisons between two yearly DA prevalence groups; 100% DA prevalence (all bowhead whales tested positive for DA) and < 100 % DA prevalence (0 – 93% tested positive for DA) in fall harvested bowhead whales (2004 – 2022). OW anomalies are calculated by dividing monthly OW areas (km^2) by the monthly baseline areas (km^2), resulting in a unitless anomaly equating baseline OW area values to 1. In all months, OW was significantly higher in years with 100% prevalence (n = 56 whales sampled across Y = 7 independently sampled years) compared to years with < 100% prevalence (n = 149 whales sampled over Y = 12 independently sampled years). EMM values were based on linear models that weighted differences in number of whales tested each year.

Month	DA Group	EMM Anomaly	Std. Error	Lower 95% CI	Upper 95% CI	Degrees of Freedom (df)	T ratio	P value
June	<100%	0.71	0.07	0.57	0.86	203	-16.92	1.17e-40
	100%	3.09	0.12	2.86	3.33	203		
July	<100%	0.93	0.05	0.83	1.03	203	-12.43	9.73e-27
	100%	2.13	0.08	1.97	2.29	203		
August	<100%	1.42	0.05	1.32	1.52	203	-8.68	1.32e-15
	100%	2.27	0.08	2.10	2.43	203		
September	<100%	1.56	0.04	1.48	1.65	203	-5.23	4.11e-07
	100%	1.97	0.07	1.84	2.11	203		

Table S4. Cruises that collected sediment samples used to produce the composite *Alexandrium catenella* cyst map (Fig. 1d). Only samples collected north of 65°N are included in this summary and map.

**Cruise ID**	**Vessel**	**Dates**	***n***
HLY1801	USCGC Healy	8/8/2018 – 8/24/2018	43
HLY1803	USCGC Healy	10/25/2018 – 11/18/2018	26
HLY1901	USCGC Healy	8/2/2019 – 8/23/2019	43
NW19-01	Northwest Explorer	8/30/2019 – 9/16/2019	5
OS19-01 (Leg 2, Leg 3)	Ocean Starr	8/25/2019 – 10/2/2019	38
DBO2020	R/V Norseman II	10/6/2020 – 10/19/2020	19
SKQ2020-14S	R/V Sikuliaq	10/15/2020 – 11/19/2020	45
SKQ2021-15S	R/V Sikuliaq	11/8/2021 – 11/16/2021	13
NW22-02	Northwest Explorer	9/1/2022 – 9/16/2022	1
NRS2022-01S	R/V Norseman II	7/19/2022 – 8/15/2022	83
NRS2022-02S	R/V Norseman II	8/17/2022 – 9/6/2022	7
SKQ2022-15S	R/V Sikuliaq	11/1/2022 – 12/3/2022	64

Table S5. July sea surface temperature (SST, °C) records for the Beaufort Sea from the NOAA OI SST V2 High Resolution Dataset data provided by the NOAA PSL, Boulder, Colorado, USA, from their website at https://psl.noaa.gov for years relevant to baselines and bowhead whale fecal samples (1982 – 2022).

Year	July (°C)
1982	0
1983	-1.27
1984	-0.28
1985	-0.84
1986	-0.54
1987	1.31
1988	0.21
1989	0.66
1990	0.76
1991	-1.4
1992	0.16
1993	2.18
1994	-0.67
1995	1.01
1996	0.56
1997	0.68
1998	3.1
1999	-0.12
2000	-0.4
2001	-0.69
2002	-0.3
2003	0.8
2004	1.5
2005	0.49
2006	0.05
2007	2.3
2008	3.31
2009	1.07
2010	1.13
2011	1.41
2012	2.49
2013	0.73
2014	1.22
2015	0.99
2016	2.65
2017	3.08
2018	-0.21
2019	4.1
2020	0.29
2021	0.17
2022	0.99

Table S6. Open water (OW) area (km^2) data, defined as areas with sea ice concentrations ≤ 15% per pixel via satellite imagery, for the Beaufort Sea from the National Snow and Ice Data Center (NSIDC) [35] for months and years relevant for baselines (1982 – 2011) and bowhead whale fecal samples (2004 – 2022).

Year	June (km^2)	July (km^2)	August (km^2)	September (km^2)
1982	54875	184248	217752	311122
1983	11757	73865	120345	78994
1984	1806	35753	76258	121984
1985	17516	37038	50537	87535
1986	6534	30781	135193	211530
1987	51615	235510	315755	262856
1988	69337	161913	153398	176982
1989	84	20614	162868	240376
1990	15755	103092	171641	206355
1991	29590	31644	35067	68711
1992	0	50094	112092	132028
1993	94538	248206	282227	353840
1994	2627	19416	127870	167486
1995	63750	187065	295325	304053
1996	208	39993	101566	70508
1997	23633	108406	220815	318143
1998	252826	418221	475256	534941
1999	11436	61904	249803	329972
2000	0	8275	159420	282115
2001	207	30839	80325	205051
2002	7682	34925	144057	436173
2003	16169	95819	186884	344444
2004	37067	124210	268167	375031
2005	87740	117477	169881	239717
2006	249	22301	157218	288265
2007	21936	184085	313746	473746
2008	113973	408089	636154	621625
2009	24438	148375	302657	456117
2010	108353	231133	372609	589278
2011	34712	245018	424176	614141
2012	125297	322061	705826	825606
2013	461	69023	281194	292654
2014	28516	222462	296431	404777
2015	80077	123536	387289	654512
2016	169988	270976	631965	755694
2017	116706	249394	561128	666656
2018	18869	77805	361827	441398
2019	197535	262657	533055	632862
2020	15145	85149	258116	477581
2021	32454	108575	191646	403647
2022	594	92924	264131	510877

Table S7. Statistical results (unpaired two-sided t-tests with no *P*-value adjustments) of the monthly summer (June – September) estimated marginal mean (EMM) Beaufort Sea open water (OW) area anomaly comparisons between yearly DA prevalence groups; **(a)** < 90% (n = 134 whales sampled across Y = 11 independently sampled years) and ≥ 90% (n = 71 whales sampled across Y = 8 independently sampled years) and **(b)** <75% (n = 106 whales sampled across Y = 9 independently sampled years) and ≥75% (n = 99 whales sampled across Y = 10 independently sampled years) domoic acid (DA). OW anomalies are calculated by dividing monthly OW areas (km^2) by the monthly baseline areas (km^2), resulting in a unitless anomaly equating baseline OW area values to 1. EMM values were based on linear models that weighted differences in number of whales tested each year.

a

**Month**	**DA Group**	**EMM Anomaly**	**Std. Error**	**Lower 95% CI**	**Upper 95% CI**	**Degrees of Freedom (df)**	**T ratio**	**P value**
June	< 90%	0.79	0.10	0.60	0.99	203	-9.79	8.91e-19
	≥ 90%	2.44	0.14	2.17	2.71			
July	< 90%	1.02	0.06	0.89	1.14	203	-6.43	9.01e-10
	≥ 90%	1.72	0.09	1.54	1.89			
August	< 90%	1.50	0.06	1.38	1.61	203	-4.34	2.27e-05
	≥ 90%	1.94	0.08	1.78	2.11			
September	< 90%	1.63	0.05	1.54	1.72	203	-1.68	0.09
	≥ 90%	1.76	0.06	1.64	1.89			

b

**Month**	**DA Group**	**EMM Anomaly**	**Std. Error**	**Lower 95% CI**	**Upper 95% CI**	**Degrees of Freedom (df)**	**T ratio**	**P value**
June	<74%	0.92	0.13	0.67	1.18	203	-4.96	1.51e-06
	≥ 75%	1.84	0.13	1.57	2.10			
July	<74%	1.01	0.08	0.87	1.16	203	-4.66	5.64e-06
	≥ 75%	1.52	0.08	1.37	1.67			
August	<74%	1.49	0.07	1.36	1.63	203	-3.24	0.001
	≥ 75%	1.82	0.07	1.68	1.96			
September	<74%	1.71	0.05	1.61	1.81	203	0.91	0.36
	≥ 75%	1.64	0.05	1.54	1.75			

Table S8: Statistical results (unpaired two-sided t-tests with no *P*-value adjustments) of the monthly summer (June – September) estimated marginal mean (EMM) Beaufort Sea open water (OW) area anomaly comparisons between two yearly STX prevalence groups; 100% STX prevalence (all bowhead whales tested positive for STX) and < 100 % STX prevalence (44 – 94% tested positive for STX) in fall harvested bowhead whales (2004 – 2022). OW anomalies are calculated by dividing monthly OW areas (km^2) by the monthly baseline areas (km^2), resulting in a unitless anomaly equating baseline OW area values to 1. In all months, OW was significantly higher in years with 100% prevalence (n = 101 whales sampled across Y = 10 years) compared to years with < 100% prevalence (n = 104 whales sampled across Y = 9 independently sampled years). EMM values were based on linear models that weighted differences in number of whales tested each year.

Month	STX Group	EMM Anomaly	Std. Error	Lower 95% CI	Upper 95% CI	Degrees of Freedom (df)	T ratio	P value
June	<100%	1.11	0.13	0.85	1.37	203	-2.70	0.008
	100%	1.63	0.14	1.36	1.89	203		
July	<100%	0.99	0.08	0.84	1.14	203	-5.04	1.01e-06
	100%	1.53	0.08	1.38	1.69	203		
August	<100%	1.44	0.07	1.30	1.57	203	-4.38	1.87e-05
	100%	1.87	0.07	1.73	2.01	203		
September	<100%	1.44	0.05	1.35	1.53	203	-7.32	5.56e-12
	100%	1.92	0.05	1.83	2.01	203		

Table S9: Arctic Data Center DOI links for Barrow mooring data (2002-2022).

AON Mooring deployment years	Arctic Data Center DOI link
2002-2003	https://arcticdata.io/catalog/view/doi%3A10.18739%2FA24T6F334
2003-2004	https://arcticdata.io/catalog/view/doi%3A10.18739%2FA2222R59X
2005-2006	https://arcticdata.io/catalog/view/doi:10.18739/A2FB4WM1G
2008-2009	https://arcticdata.io/catalog/view/doi%3A10.18739%2FA25T3G02W
2009-2010	https://arcticdata.io/catalog/view/doi%3A10.18739%2FA29W09071
2010-2011	https://arcticdata.io/catalog/view/doi%3A10.18739%2FA2GH9B984
2011-2012	https://arcticdata.io/catalog/view/doi%3A10.18739%2FA2BR8MH3H
2012-2013	https://arcticdata.io/catalog/view/doi%3A10.18739%2FA2707WP8K
2013-2014	https://arcticdata.io/catalog/view/doi%3A10.18739%2FA23775W4T
2014-2016	https://arcticdata.io/catalog/view/doi%3A10.18739%2FA29K45S73
2016-2018	https://arcticdata.io/catalog/view/doi%3A10.18739%2FA2ZG6G797
2018-2020	https://arcticdata.io/catalog/view/doi%3A10.18739%2FA2HX15S0W
2020-2022	https://arcticdata.io/catalog/view/doi:10.18739/A27659H36
