# Supplementary material for: Bowhead whale faeces link increasing algal toxins in the Arctic to ocean warming
Source: Nature. 2025 Jul 9;644(8077):693–8. doi: 10.1038/s41586-025-09230-5 (PMC12367538; doi:10.1038/s41586-025-09230-5)
Supplement: Supplementary file 2 — Reporting Summary [file 41586_2025_9230_MOESM2_ESM.pdf]

nature portfolio

Corresponding author(s): Kathi Lefebvre

Last updated by author(s): May 13, 2025

Reporting Summary

Nature Portfolio wishes to improve the reproducibility of the work that we publish. This form provides structure for consistency and transparency in reporting. For further information on Nature Portfolio policies, see our Editorial Policies and the Editorial Policy Checklist.

Please do not complete any field with "not applicable" or n/a. Refer to the help text for what text to use if an item is not relevant to your study.
For final submission: please carefully check your responses for accuracy; you will not be able to make changes later.

Statistics

For all statistical analyses, confirm that the following items are present in the figure legend, table legend, main text, or Methods section.

n/a	Confirmed	
☐	☒	The exact sample size (*n*) for each experimental group/condition, given as a discrete number and unit of measurement
☐	☒	A statement on whether measurements were taken from distinct samples or whether the same sample was measured repeatedly
☐	☒	The statistical test(s) used AND whether they are one- or two-sided<br>*Only common tests should be described solely by name; describe more complex techniques in the Methods section.*
☐	☒	A description of all covariates tested
☐	☒	A description of any assumptions or corrections, such as tests of normality and adjustment for multiple comparisons
☐	☒	A full description of the statistical parameters including central tendency (e.g. means) or other basic estimates (e.g. regression coefficient) AND variation (e.g. standard deviation) or associated estimates of uncertainty (e.g. confidence intervals)
☐	☒	For null hypothesis testing, the test statistic (e.g. *F*, *t*, *r*) with confidence intervals, effect sizes, degrees of freedom and *P* value noted<br>*Give P values as exact values whenever suitable.*
☒	☐	For Bayesian analysis, information on the choice of priors and Markov chain Monte Carlo settings
☒	☐	For hierarchical and complex designs, identification of the appropriate level for tests and full reporting of outcomes
☐	☒	Estimates of effect sizes (e.g. Cohen's *d*, Pearson's *r*), indicating how they were calculated

Our web collection on statistics for biologists contains articles on many of the points above.

Software and code

Policy information about availability of computer code

Data collection

No software was used for data collection.

Data analysis

Comparisons of environmental data (open water area anomalies) and algal toxin prevalence groupings of whales was done using freely available computer software. Analysis was done using software programs R (version 4.4.2) and R studio (version 2024.09.1+394). Specifically we used the following R packages: "lme4" (v. 1.1-25.5) for constructing linear models, estimated marginal means were generated and compared using pairwise comparisons (unpaired t-tests) among whale groupings using "emmeans" (v. 1.10.5), and Pearson correlation analysis of June open water anomalies and July sea surface temperature anomalies was done using the package "ggpubr" (v. 0.6.0). The calculations of heat flux and wind/sea level pressure composites were done using MATLAB 2024b. All software programs and packages are properly cited in the manuscript.

For manuscripts utilizing custom algorithms or software that are central to the research but not yet described in published literature, software must be made available to editors and reviewers. We strongly encourage code deposition in a community repository (e.g. GitHub). See the Nature Portfolio guidelines for submitting code & software for further information.

Data

Policy information about availability of data

All manuscripts must include a data availability statement. This statement should provide the following information, where applicable:

- Accession codes, unique identifiers, or web links for publicly available datasets
- A description of any restrictions on data availability
- For clinical datasets or third party data, please ensure that the statement adheres to our policy

Bowhead whale fecal algal toxin concentrations (DA and STX) and whale collection dates are available in the supplementary materials (Table S1). Alexandrium catenella cyst data for 2018 – 2020 can be found at the Arctic Data Center database (https://doi:10.18739/A2RF5KG8J , https://doi:10.18739/A2Q814V0P)22. Alexandrium cell density data are included in the supplementary materials (Table S3). Hydrographic and velocity data from the mooring near Barrow Canyon were retrieved from the Arctic Observing Network Data Center and the DOI links for 2002-2022 are given in Supplementary Table S948,49. Wind velocity and sea level pressure data were provided by the European Centre for Medium-Range Weather Forecasts (ECMWF) ERA5 reanalysis dataset (https://cds.climate.copernicus.eu/datasets/reanalysis-era5-single-levels)50. July sea surface temperature (SST) data and open water area data during the summer months for the Beaufort Sea are provided in the supplementary material (Table S4 and Table S5, respectively). SST data from 1900 – 2023 for the Bering, Chukchi, and Beaufort Seas were obtained from the NOAA Extended Reconstructed SST V5 data provided by the NOAA PSL, Boulder, Colorado, USA, from this link https://psl.noaa.gov/data/gridded/data.noaa.ersst.v5.html 34. The sea ice extent data for Bering, Chukchi, and Beaufort Seas (1979 – 2024) were acquired from the National Snow and Ice Data Center at the following link https://noaadata.apps.nsidc.org/NOAA/G02135/seaice_analysis/N_Sea_Ice_Index_Regional_Daily_Data_G02135_v3.0.xlsx35.

Research involving human participants, their data, or biological material

Policy information about studies with human participants or human data. See also policy information about sex, gender (identity/presentation), and sexual orientation and race, ethnicity and racism.

Reporting on sex and gender	No human samples
Reporting on race, ethnicity, or other socially relevant groupings	No human samples
Population characteristics	No human samples
Recruitment	No human samples
Ethics oversight	No human samples

Note that full information on the approval of the study protocol must also be provided in the manuscript.

Field-specific reporting

Please select the one below that is the best fit for your research. If you are not sure, read the appropriate sections before making your selection.

☐ Life sciences ☐ Behavioural & social sciences ☒ Ecological, evolutionary & environmental sciences

For a reference copy of the document with all sections, see nature.com/documents/nr-reporting-summary-flat.pdf

Ecological, evolutionary & environmental sciences study design

All studies must disclose on these points even when the disclosure is negative.

Study description	Bowhead fecal samples were collected from landed whales during indigenous fall harvest seasons over 19 years in order to measure the presence of algal toxins in Arctic food webs.
Research sample	Feces extracted from the bowel of harvested whales.
Sampling strategy	All samples used in this study were comparable in terms of collection techniques and time of year (Fall harvest season).
Data collection	Fecal samples were analyzed for the presence of the algal toxins saxitoxin and domoic acid.
Timing and spatial scale	Bowhead whales were havested from the Beaufort Sea from August to October from 2004 to 2022.
Data exclusions	Not applicable
Reproducibility	Not applicable
Randomization	Not applicable

Blinding	Not applicable

Did the study involve field work? ☒ Yes ☐ No

Field work, collection and transport

Field conditions	Arctic conditions over 19 years of whale harvesting.
Location	Whales were landed at Utqiaġvik, Alaska.
Access & import/export	All samples were collected in collaboration with the North Slope Borough Department of Wildlife Management as part of their whale health monitoring program.
Disturbance	Traditional hunting practices were used for harvesting whales. Fecal samples were collected post mortem.

Reporting for specific materials, systems and methods

We require information from authors about some types of materials, experimental systems and methods used in many studies. Here, indicate whether each material, system or method listed is relevant to your study. If you are not sure if a list item applies to your research, read the appropriate section before selecting a response.

Materials & experimental systems

n/a	Involved in the study
☒	☐ Antibodies
☒	☐ Eukaryotic cell lines
☒	☐ Palaeontology and archaeology
☐	☒ Animals and other organisms
☒	☐ Clinical data
☒	☐ Dual use research of concern
☒	☐ Plants

Methods

n/a	Involved in the study
☒	☐ ChIP-seq
☒	☐ Flow cytometry
☒	☐ MRI-based neuroimaging

Animals and other research organisms

Policy information about studies involving animals; ARRIVE guidelines recommended for reporting animal research, and Sex and Gender in Research

Laboratory animals	Laboratory animals were not used in this study.
Wild animals	No live wild animals were used in this study. Fecal samples were collected opportunistically post mortem from bowhead whales harvested for subsistence uses by Native tribal communities.
Reporting on sex	Sex was not reported.
Field-collected samples	Bowhead whale fecal samples were collected post mortem during harvest processing of whales by indigenous hunters and North Slope Borough Department of Wildlife Management staff.
Ethics oversight	Ethics oversight was not needed. Fecal samples were collected from deceased whales under National Marine Fisheries permit number 17350-00

Note that full information on the approval of the study protocol must also be provided in the manuscript.

Plants

Seed stocks	NA
Novel plant genotypes	NA
Authentication	NA

This checklist template is licensed under a Creative Commons Attribution 4.0 International License, which permits use, sharing, adaptation, distribution and reproduction in any medium or format, as long as you give appropriate credit to the original author(s) and the source, provide a link to the Creative Commons license, and indicate if changes were made. The images or other third party material in this article are included in the article's Creative Commons license, unless indicated otherwise in a credit line to the material. If material is not included in the article's Creative Commons license and your intended use is not permitted by statutory regulation or exceeds the permitted use, you will need to obtain permission directly from the copyright holder. To view a copy of this license, visit http://creativecommons.org/licenses/by/4.0/
